# Simultaneous Modeling of Young’s Modulus, Yield Stress, and Rupture Strain of Gelatin/Cellulose Acetate Microfibrous/Nanofibrous Scaffolds Using RSM

**DOI:** 10.3389/fbioe.2021.718718

**Published:** 2021-09-13

**Authors:** Alireza Barazesh, Mahdi Navidbakhsh, Ali Abouei Mehrizi, Mojtaba Koosha, Sajad Razavi Bazaz, Tianduo Li

**Affiliations:** ^1^ Tissue Engineering and Biological Systems Research Laboratory, School of Mechanical Engineering, Iran University of Science and Technology, Tehran, Iran; ^2^ Department of Life Science Engineering, Faculty of New Sciences and Technologies, University of Tehran, Tehran, Iran; ^3^ Shandong Provincial Key Laboratory of Molecular Engineering, School of Chemistry and Chemical Engineering, Qilu University of Technology (Shandong Academy of Sciences), Jinan, China; ^4^ School of Biomedical Engineering, University of Technology Sydney, Sydney, NSW, Australia

**Keywords:** electrospinning, fiber alignment, gelatin, cellulose acetate, mechanical properties, rsm

## Abstract

Electrospinning is a promising method to fabricate bioengineered scaffolds, thanks to utilizing various types of biopolymers, flexible structures, and also the diversity of output properties. Mechanical properties are one of the major components of scaffold design to fabricate an efficacious artificial substitute for the natural extracellular matrix. Additionally, fiber orientations, as one of the scaffold structural parameters, could play a crucial role in the application of fabricated fibrous scaffolds. In this study, gelatin was used as a highly biocompatible polymer in blend with cellulose acetate (CA), a polysaccharide, to enhance the achievable range of mechanical characteristics to fabricated fibrous electrospun scaffolds. By altering input variables, such as polymers concentration, weight ratio, and mandrel rotation speed, scaffolds with various mechanical and morphological properties could be achieved. As expected, the electrospun scaffold with a higher mandrel rotation speed shows higher fiber alignment. A wide range of mechanical properties were gained through different values of polymer ratio and total concentration. A general improvement in mechanical strength was observed by increasing the concentration and CA content in the solution, but contradictory effects, such as high viscosity in more concentrated solutions, influenced the mechanical characteristics as well. A response surface method was applied on experimental results in order to describe a continuous variation of Young’s modulus, yield stress, and strain at rupture. A full quadratic version of equations with the 95% confidence level was applied for the response modeling. This model would be an aid for engineers to adjust mandrel rotation speed, solution concentration, and gelatin/CA ratio to achieve desired mechanical and structural properties.

## Introduction

Tissue engineering is becoming a promising method to replace conventional transplants which face several limitations, including the lack of donors and insufficient adaption with the immune system of the patient. Tissue engineers are making every effort to design and build different types of tissues to replace the damaged ones (Devices, 2000). Various types of artificial tissues have been designed by tissue engineers, such as the bone (Al-Munajjed et al., 2009; Al-Munajjed and O’Brien, 2009; Brown et al., 2015; Atila et al., 2016), the cartilage (Chang et al., 2003; Chang et al., 2006; Liao et al., 2007; Kang et al., 2016), the nerve tissue (Chang et al., 2003; Chang et al., 2006; Liao et al., 2007; Kang et al., 2016), the vascular tissue (Crompton et al., 2007; Binan et al., 2014; Lee et al., 2017a), and the artificial skin (Brady et al., 2008; Schumann et al., 2009; Lee et al., 2017b) or even healing diabetic wounds by the aid of tissue engineering principles (Tangsadthakun et al., 2006; Wang et al., 2006; Chen et al., 2009; Tan et al., 2012; Wittmann et al., 2015; Wang et al., 2016).

Scaffolds are one of the vital parts of the designed tissues. They should meet the characteristics of the natural extracellular matrix (ECM), including biocompatibility, biodegradability, bioactivity, and mechanical properties. Researchers are investigating the determination of scaffold mechanical properties to mimic the original ECM so that the whole designed tissue would work efficiently. Kalakonda et al. (Kalakonda et al., 2017) improved the poly (glycerol sebacate)/poly (ε-caprolactone) fibrous scaffold strength by coating the fibers with silver. Niaza et al. (Niaza et al., 2017) designed and modified polylactide-based scaffolds to gain interesting mechanical characteristics with the addition of microparticles/nanoparticles of hydroxyapatite. Yuan et al. (Yuan et al., 2017) put much effort into studying pressure characteristic alteration during degradation of a hydrogel scaffold.

Various methods have been applied to build scaffolds; each has its own benefits and limitations. Among these methods, 3D bioprinting (Brady et al., 2008; Merceron et al., 2015; Kang et al., 2016; Huang et al., 2017), stereolithography (Lee et al., 2017b; Guillaume et al., 2017), and electrospinning are the most popular ones. Electrospinning is known for its simplicity and ability to fabricate a wide range of mechanical and structural fibrous scaffolds, which could be exploited for different types of tissues to be regenerated. The skin (Atila et al., 2015), neural tissue (Binan et al., 2014), bone (Yao et al., 2017), and cartilage (Reboredo et al., 2016) are examples of various scaffolds generated by electrospinning.

There are some variables in the electrospinning procedure that would be able to influence the scaffold characteristics. Solution properties, mostly solution concentration and polymer weight ratio, are crucial parameters which determine whether the solution is able to be spun or not. Applied voltage and flow rate of the system are the other effective parameters on the fiber morphology. The distance between the tip of the syringe and the mandrel, in addition to the mandrel rotation speed, could also affect the characteristics of the fabricated scaffold. Techniques such as adding nanoparticles to the solution could improve the mechanical properties of the scaffold. However, the determination of how these process parameters could affect mechanical properties would be a precious guide to reduce the number of experiments and the cost of design to achieve the desired mechanical properties. Endeavors have been conducted to clarify the effect of different variables on output properties, but most of these research studies focused on defining only one of these variables on a single property of the scaffold (Lee et al., 2017a), (Fong et al., 1999)– (Cells and Hall, 2011). Holding mechanical attitude, although Vatankhah et al. (Vatankhah et al., 2014a) modeled the electrospinning process with an artificial neural network to predict the elastic modulus of the scaffolds as a function of three input variables, yield stress and strain at the rupture of the scaffold are needed to study either.

Gelatin is an organic polymer whose high biocompatibility and bioactivity are the reasons to use it widely for tissue engineering purposes (Fong et al., 1999; Megelski et al., 2002; Chang et al., 2003; Wang et al., 2006). Cellulose acetate (CA) is also another organic polymer that has a plant origin. Cellulose-based polymers are commonly employed to gain better mechanical properties. Blend of CA with other bioactive biopolymers could satisfy both bioactivity and mechanical properties of the scaffold (Cells and Hall, 2011; Hafidz and Yaakob, 2011; Vatankhah et al., 2014a; Nadim et al., 2017).

Since numerous factors affect the mechanical properties of a fabricated tissue, including materials and the fabrication technology, defining a relationship between these two items is satisfactory. Design of experiment (DOE), as a powerful toolbox, was utilized in order to gain a new insight into the effect of parameters involved in an experiment and widely used in the literature for various kinds of applications (Liu and Hsieh, 2002), (Goetz et al., 2016). The response surface methodology (RSM) explores the relationship between variables and responses in a continuous manner, which is a dexterous toolbox for further analysis of the results. Indeed, a nonlinear relationship between variables and responses, which is the output of RSM, can be used as a reference for analysis of different conditions of the variables; in contrast to conventional methods, the interaction among process variables can be determined by statistical techniques (Vatankhah et al., 2014b). This method was first introduced by George Box (Cheng et al., 2017) and developed by Box and Wilson (Box et al., 1978; Box and Wilson, 1992; Anderson and Whitcomb, 2010; Bazaz et al., 2018; Mollajan et al., 2018). Since modeling the mechanical properties of a fabricated tissue with its affected parameters is uninvestigated, to date, using this method can give a new insight into the relationship between factors and results and further discussions can be obtained as a result. Although mechanical characteristics of scaffolds have been evaluated so far (Lee et al., 2017a), (Fong et al., 1999)- (Vatankhah et al., 2014a), mainly attempts were made on modeling a single mechanical characteristic (Vatankhah et al., 2014a).

In this study, we try to develop a model which predicts three mechanical characteristics of tissues, Young’s modulus, yield stress, and rupture strain, simultaneously instead of predicting them individually by knowing the solution and fabrication properties of gelatin/CA electrospun scaffolds. To endorse our model accuracy, we check the analysis of our data and model efficiency.

## Experimental and Modeling

### Materials

Gelatin (Gel) type A (300 Bloom) from the porcine skin was purchased from Sigma-Aldrich; CA, average M_w_ = 100,000 and acetyl group 39.8%, was purchased from Acros (United States); and glacial acetic acid was purchased from Merck (Germany).

### Electrospinning

Solutions were made up of Gel and CA with concentrations of 12, 13, 15, and 17% (w/v), dissolved in pure glacial acetic acid with polymer weight ratios (Gel:CA) of 100:0, 90:10, 80:20, and 70:30, respectively. The solutions were heated and stirred for 10 h to substantiate the complete dissolving. The mandrel rotation speed of the electrospinning device was set to 200, 400, 800, and 1,200 rpm. Experiments were designed using the Taguchi algorithm for three parameters with four levels which reduce the number of experiments from 64 to 16. This set of experiments will lead us to study the mechanical behavior of Gel/CA scaffolds as a function of solution concentration, polymer weight ratio, and mandrel rotation speed. The set of experiments which we need based on the Taguchi algorithm is listed in Table 1.

**TABLE 1:** Parameters’ values based on the Taguchi method.

Sample number	Concentration (w/v)	Gelatin content (%)	Mandrel rotation speed (rpm)
1	12	80	200
2	12	90	400
3	12	70	800
4	12	100	1,200
5	13	80	400
6	13	90	200
7	13	70	1,200
8	13	100	800
9	15	80	800
10	15	90	1,200
11	15	70	200
12	15	100	400
13	17	80	1,200
14	17	90	800
15	17	70	400
16	17	100	200

Polymeric solutions were loaded into a 1 ml syringe and electrospun from 27G blunted stainless-steel needles. An applied voltage of 15–19 kV and a flow rate of 3–5 ml h^−1^ were set for each scaffold for the sake of achieving beadless fibers. The polymer solution was electrospun as far as reaching a suitable fibrous membrane with a thickness of 180–400 µm. To obtain a homogeneous thickness along the mandrel axis, the syringe and the needle attached to the syringe pump were moving horizontally with a constant speed. The distance between the needle tip and the mandrel was set at 13 cm for all samples, and the experiment took place under room conditions with a temperature of 27 °C and a humidity of 40%.

### Mechanical Characterization

The fabricated electrospun scaffolds were kept at room temperature for 15 days so as to uphold perfect solvent vaporization. The specimens with 30 mm in length and 5 mm in width were glued to a paper frame, designed to place the specimens into the mechanical testing machine, resulting in a gauge length of 20 mm for the specimens. Preparing dog bone-shaped samples from the fabricated scaffolds which have Gel as the dominant element is hard to handle; thus, we decided to use rectangular samples. For the sake of assuring that rectangular specimens will not affect the results, any result of the specimens which were broken at their edges was excluded. The thickness of each scaffold was measured with a digital micrometer. A uniaxial tensile test was conducted on each scaffold. A tabletop Santam STM-1 bench test machine was employed with a 6 kgf load cell under a load rate of 1 mm/min. At least three samples from each scaffold were tested. In order to measure the rupture strain for the sample, all tests were conducted until the rupture of the specimen. Elastic modulus, yield stress, and the rupture strain were measured through the recommended protocol by the manufacturer of the testing machine and software (SANTAM).

### Scanning Electron Microscopy

Structural and morphological characteristics of the fabricated scaffolds were determined by the aid of scanning electron microscopy (SEM) (Tescan Vega-II, Czech Republic) at an accelerating voltage of 30 kV on the gold-coated samples. Fiber orientation was determined by utilizing ImageJ software, a public domain Java image processing program inspired by NIH Image. ImageJ can display, edit, analyze, process, save, and print 8-bit, 16-bit, and 32-bit images, in addition to calculating the area and pixel value statistics of user-defined selections. To maintain the randomness of the evaluation, three images from different parts of each sample were captured and analyzed.

### Fiber Alignment Quality

In order to attain a better view of fiber alignment, after measuring the angles of the fibers, related angles to the dominant direction were also calculated and two parameters α and *β* were defined to represent the quality of fiber alignment in a single scaffold. α is the ratio of the fibers with less than “30 absolute degrees” deviation from the dominant direction as represented in Eq. 1.

$$\alpha =\frac{number\;of\;fibers\;with\;less\;than\;30\;degrees\;deviation\;of\;dominant\;direction}{all\;of\;the\;detected\;fibers\;in\;a\;single\;scaffold\;SEM\;images\;\;\;}\times 100$$. (1)

Eq. 2 shows how the *β* value is defined, which is also the standard deviation of the related angles of the fibers from the dominant angle

$$\beta =Standard\;deviation\left\{\theta_{n}\right\},$$ (2)

where *θ*_*n*_ is the angle between each fiber related to the dominant direction. The dominant angle of each sample was the average angle of fiber angles in SEM images.

### Data Analysis

Since our data are continuous, the response surface method, a toolkit for data analysis is used to investigate the data statistically. The parameters used in this analysis are concentration, polymer weight ratio, and mandrel rotation speed. The method on which the results relied is based on an uncoded value, and the number of experiments is set to 16. In order to analyze response surface design, a full quadratic version of the equation is used, which is described in Eq. 3.

$$Y=\beta_{0}+\sum_{j=1}^{k}\beta_{j}X_{j}+\sum_{j=1}^{k}\beta_{jj}X_{j}^{2}+\sum_{i}\sum_{j\geq i}^{k}\beta_{ij}X_{i}X_{j}+e_{i}.$$ (3)

Here, a constant coefficient is described by $\beta_{0}$; the interactions of linear, quadratic, and second-order coefficients are represented by $\beta_{j}$, $\beta_{jj}$, and $\beta_{ij}$, respectively; Y is the response; the variables are $X_{i}$ and $X_{j}$; the number of studied parameters is *k*; and $e_{i}$ is the error. For the analysis of variances, the *p*-value is set as the 95% confidence level to evaluate the interaction between the identical and nonidentical variables. For strain, the optimal level of Box-Cox transformation is used with the two-sided type of confidence level, while for stress, the Box-Cox transformation is set to 0.3 with a two-sided confidence level. Furthermore, the stepwise method is applied for entering or removing the terms in the full quadratic equation of RSM. For modulus, forward selection of parameters is set, while no Box-Cox transformation with a two-sided level of confidence is elected.

## Results

### Morphological Results

**FIGURE 1:**
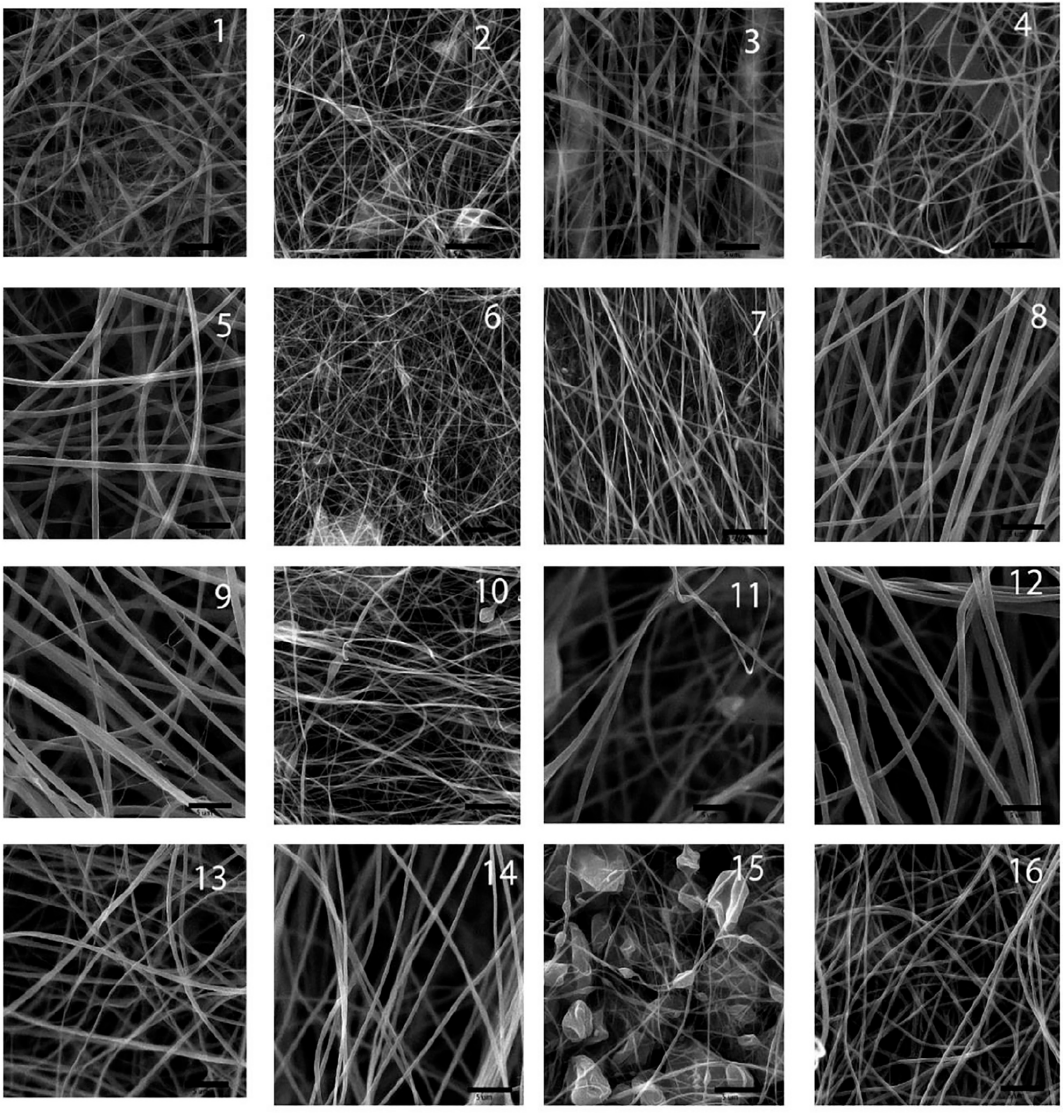
SEM images of the fabricated scaffolds.

**FIGURE 2:**
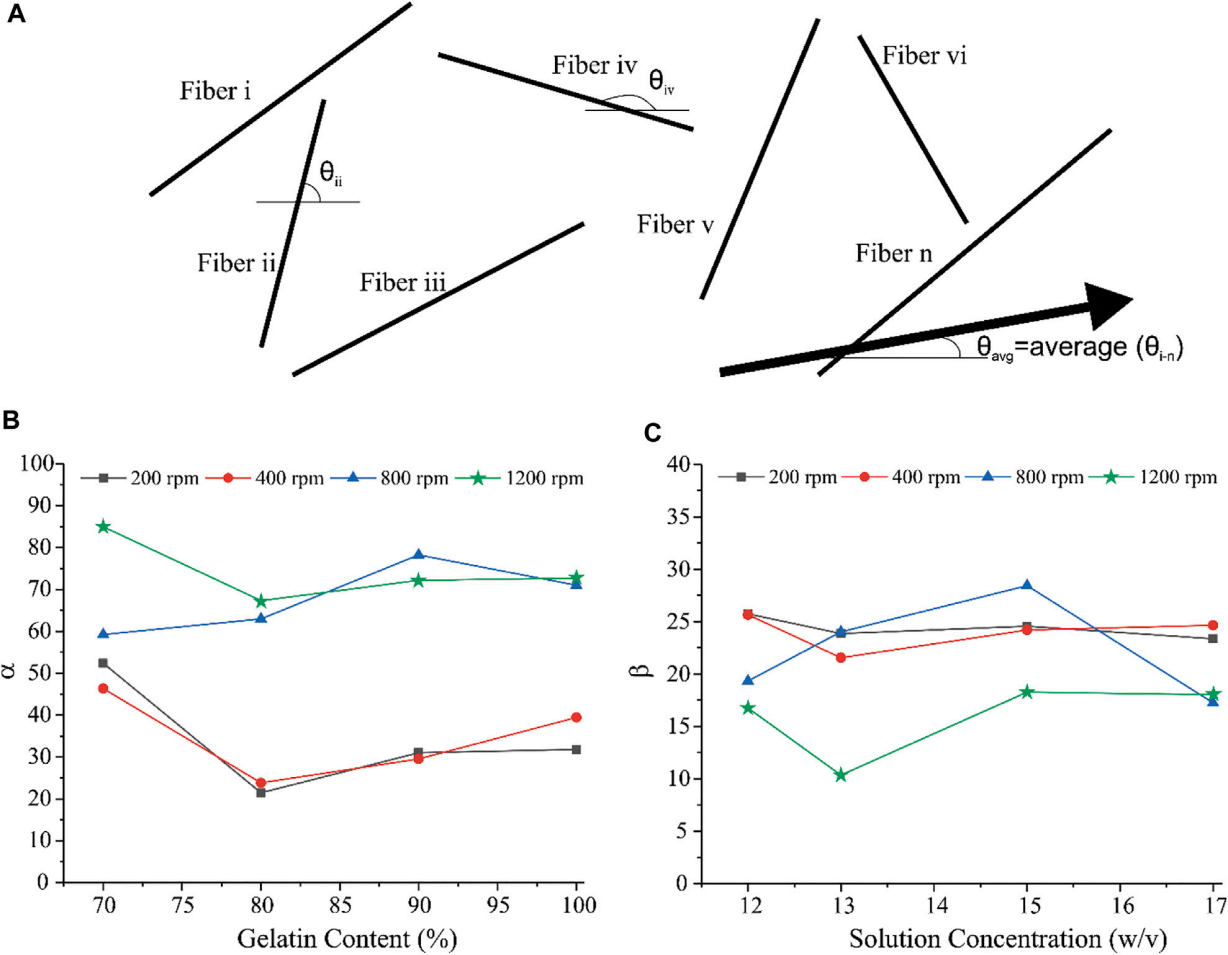
**(A)** schematic illustration of fiber alignment. **(B)** Variation of α with respect to polymer weight ratio and **(C)** variation of *ß* with respect to solution concentration.

Figure 1 illustrates the microscopic morphology of fabricated scaffolds. Table 2 represents the sample numbers and the values of alignment coefficients α and *β* based on Eqs 1, 2. As written in Tables 1, 2, with the increase of the mandrel rotation speed, parameter α is increased alongside the decrease in parameter *β*. For a better understanding of the influence of other input variables on fiber alignment, Figure 2 was prepared. Figure 2A shows how the fiber alignment was quantified. At a constant mandrel rotation speed, polymer weight ratio could affect the number of fibers close to the dominant direction. Also, the solution concentration affects the fiber distribution in the scaffolds.

**TABLE 2:** Values of fiber diameter, α, and *ß* calculated for the scaffolds.

Sample number	Fiber diameter (nm)	α	β
1	111.56 ± 46	21.43	25.747
2	123.03 ± 50	29.55	25.656
3	199.58 ± 85	54.84	19.335
4	232.77 ± 42	68.97	16.745
5	470.17 ± 239	23.81	21.546
6	93.06 ± 25	31	23.85
7	2056.65 ± 537	85	10.346
8	507.57 ± 84	60	24.024
9	781.59 ± 132	62.96	24.441
10	126.50 ± 45	72.22	18.271
11	360.63 ± 102	52.38	24.75
12	681.17 ± 189	39.47	24.194
13	372.70 ± 96	67.35	18.012
15	409.24 ± 61	46.34	17.245
14	146.53 ± 50	78.26	24.643
16	305.12 ± 53	31.82	24.37

### Mechanical Results

**FIGURE 3:**
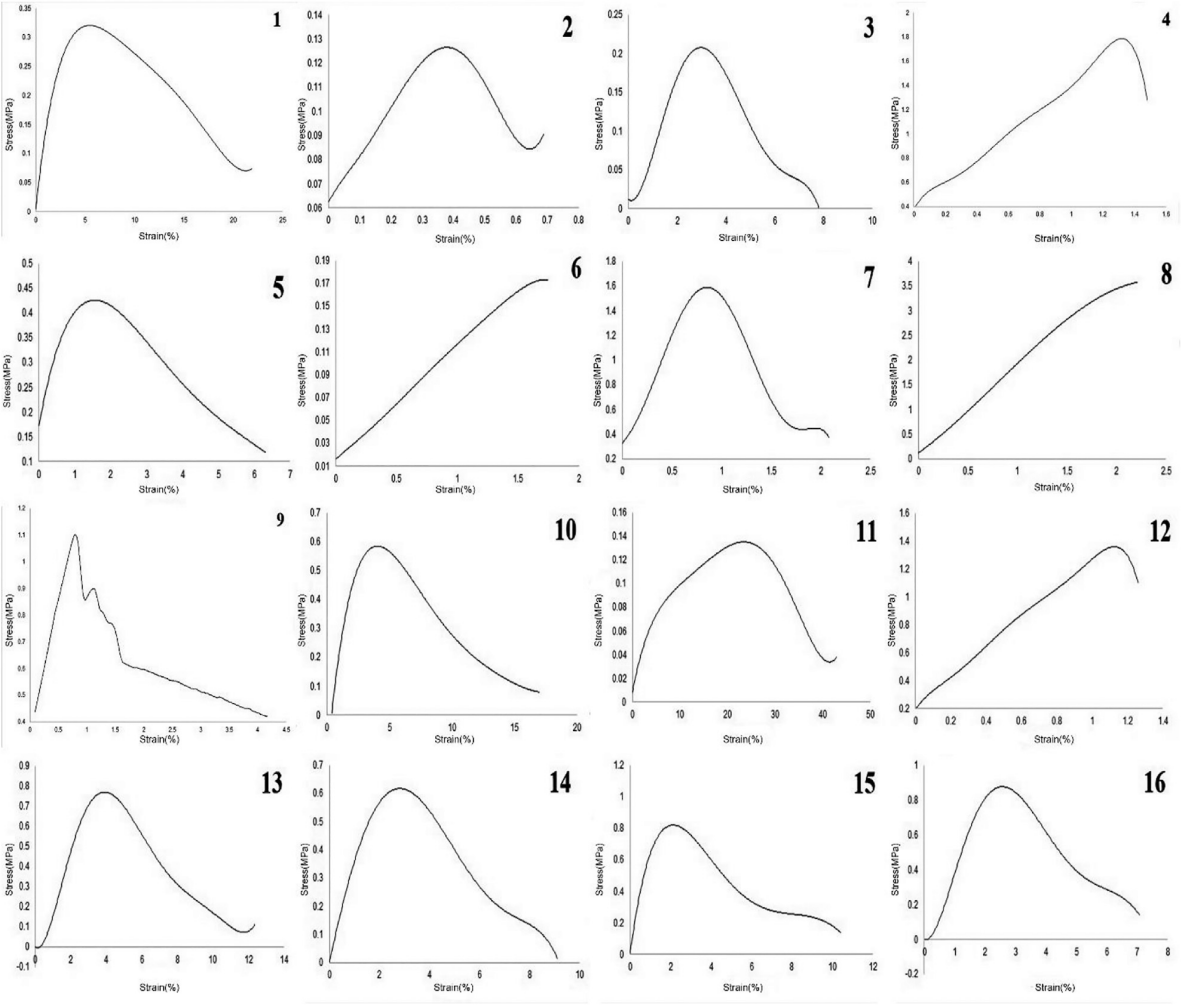
Stress–strain curve of 16 experiments in this study.

The mechanical outputs of SANTAM software are listed in Table 3, and the stress–strain curve of all experiments is shown in Figure 3. It indicates the mean average of each of the three desired parameters with their standard deviation. The results show a complicated interaction between input variables and how they influence mechanical characteristics, where none of the variable effects could be discussed without considering others.

**TABLE 3:** Mechanical properties of the scaffolds.

Sample number	Elastic modulus (MPa)	Yield stress (MPa)	Rupture strain (%)
1	4.33 ± 2.13	0.31 ± 0.12	24.62 ± 2.15
2	29.89 ± 18.87	0.09 ± 0.05	1.84 ± 0.82
3	8.72 ± 2.35	0.22 ± 0.05	8.47 ± 0.51
4	126.8 ± 76.11	2.1 ± 0.46	2.67 ± 0.49
5	60.68 ± 30.8	0.39 ± 0.09	9.65 ± 3.35
6	10.04 ± 0.48	0.17 ± 0.02	2.16 ± 0.09
7	194.36 ± 75.1	1.5 ± 0.48	4.51 ± 0.21
8	146.87 ± 44.01	3.55 ± 0.57	2.82 ± 0.83
9	183.74 ± 55.71	0.91 ± 0.04	6.4 ± 0.79
10	18.62 ± 4.47	0.61 ± 0.18	21.39 ± 4
11	0.56 ± 0.16	0.14 ± 0.02	52.56 ± 3.96
12	114.34 ± 25.19	1.28 ± 0.19	2.11 ± 0.67
13	25.35 ± 7.18	0.76 ± 0.14	18.3 ± 1.93
14	34.42 ± 1.54	0.72 ± 0.09	11.57 ± 1.33
15	45.38 ± 17.98	0.87 ± 0.24	20.83 ± 3.12
16	42.85 ± 8.54	1.09 ± 0.05	10.49 ± 2.51

### RSM Method Results

**FIGURE 4:**
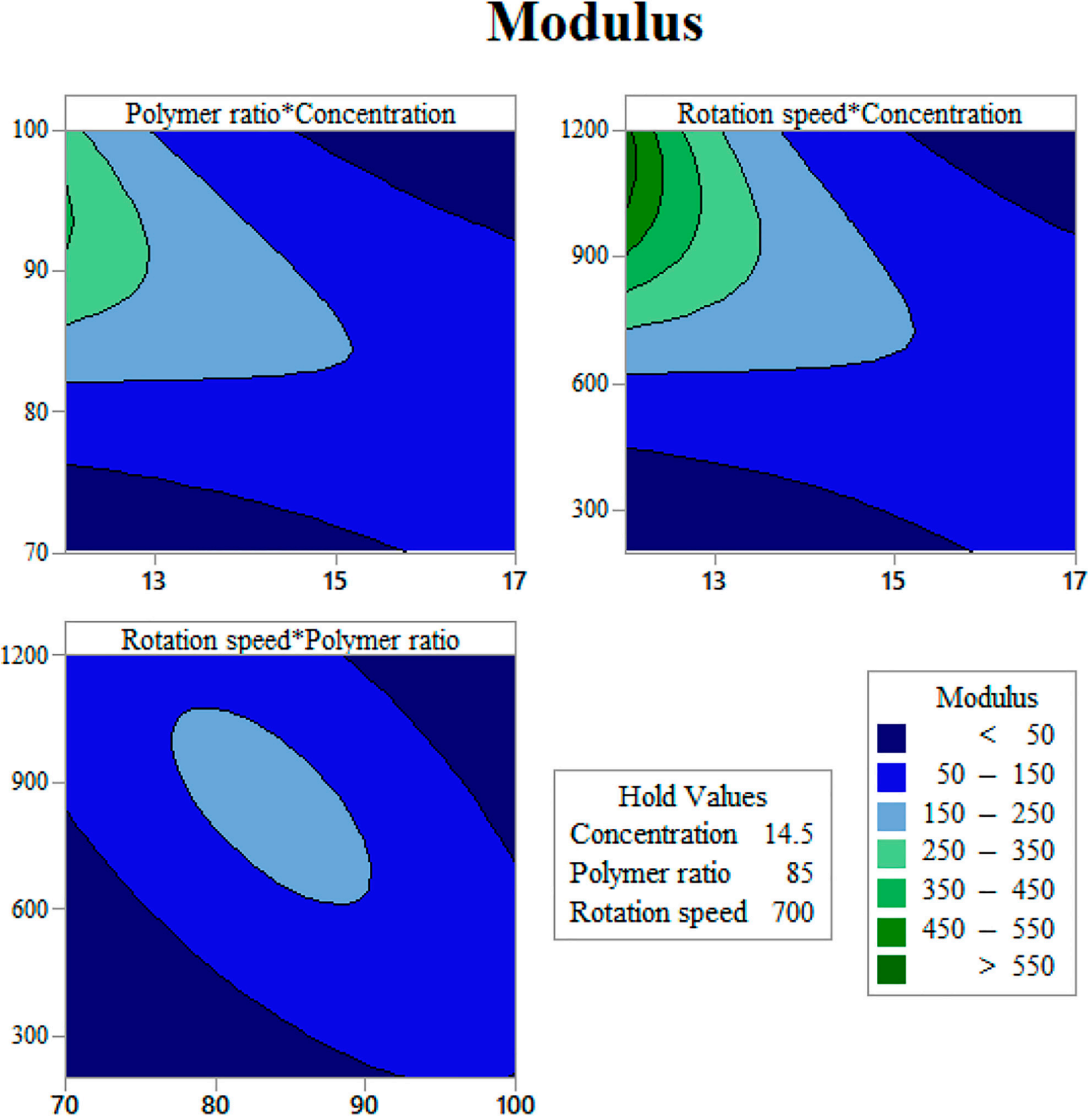
Elastic modulus contours as functions of the input variables: **(A)** gelatin content vs. concentration, **(B)** mandrel rotation speed vs. concentration, and **(C)** mandrel rotation speed vs. gelatin content.

**FIGURE 5:**
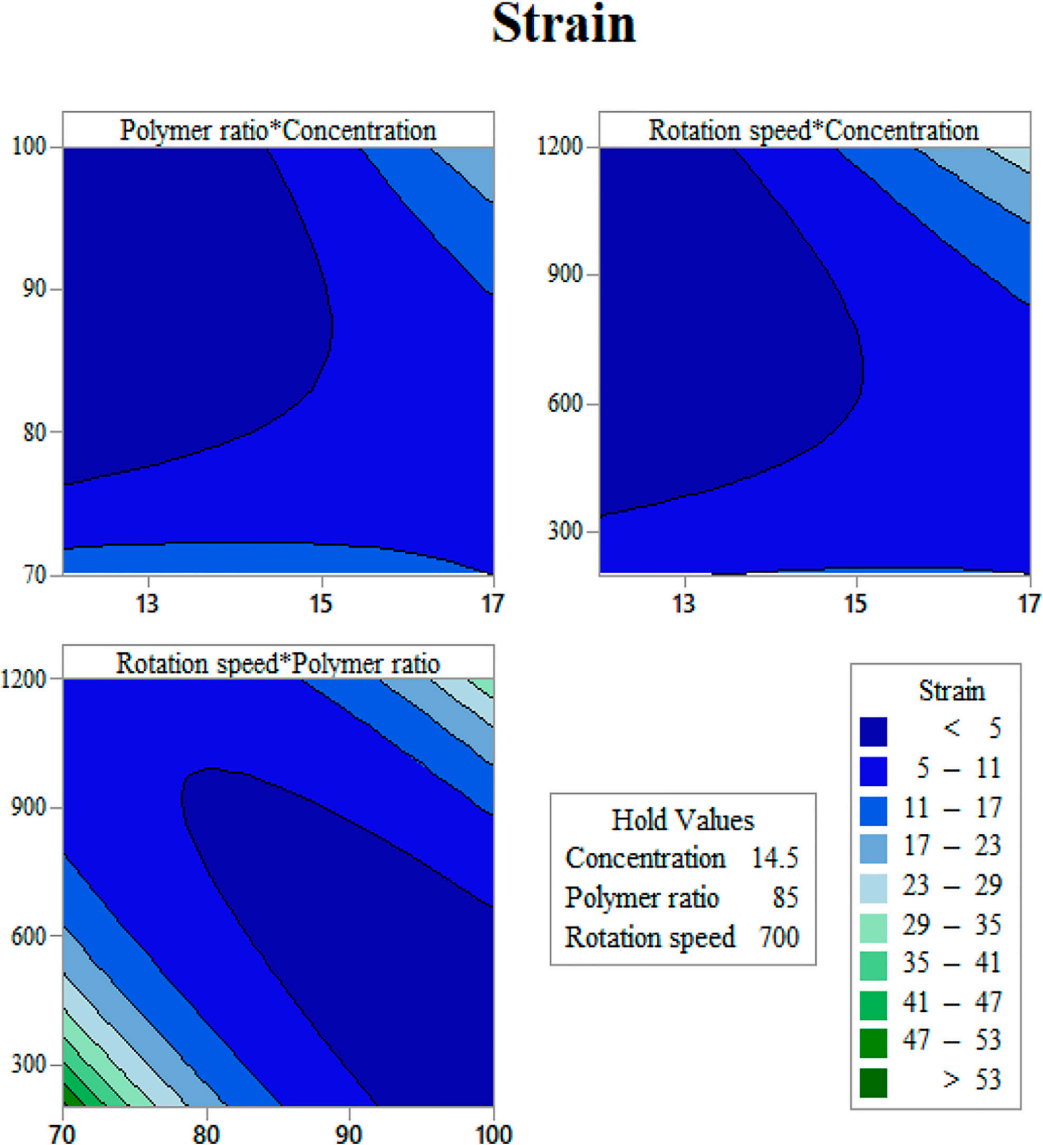
Strain at rupture contours as functions of input variables: **(A)** gelation content vs. concentration, **(B)** mandrel rotation speed vs. concentration, and **(C)** mandrel rotation speed vs gelatin content.

**FIGURE 6:**
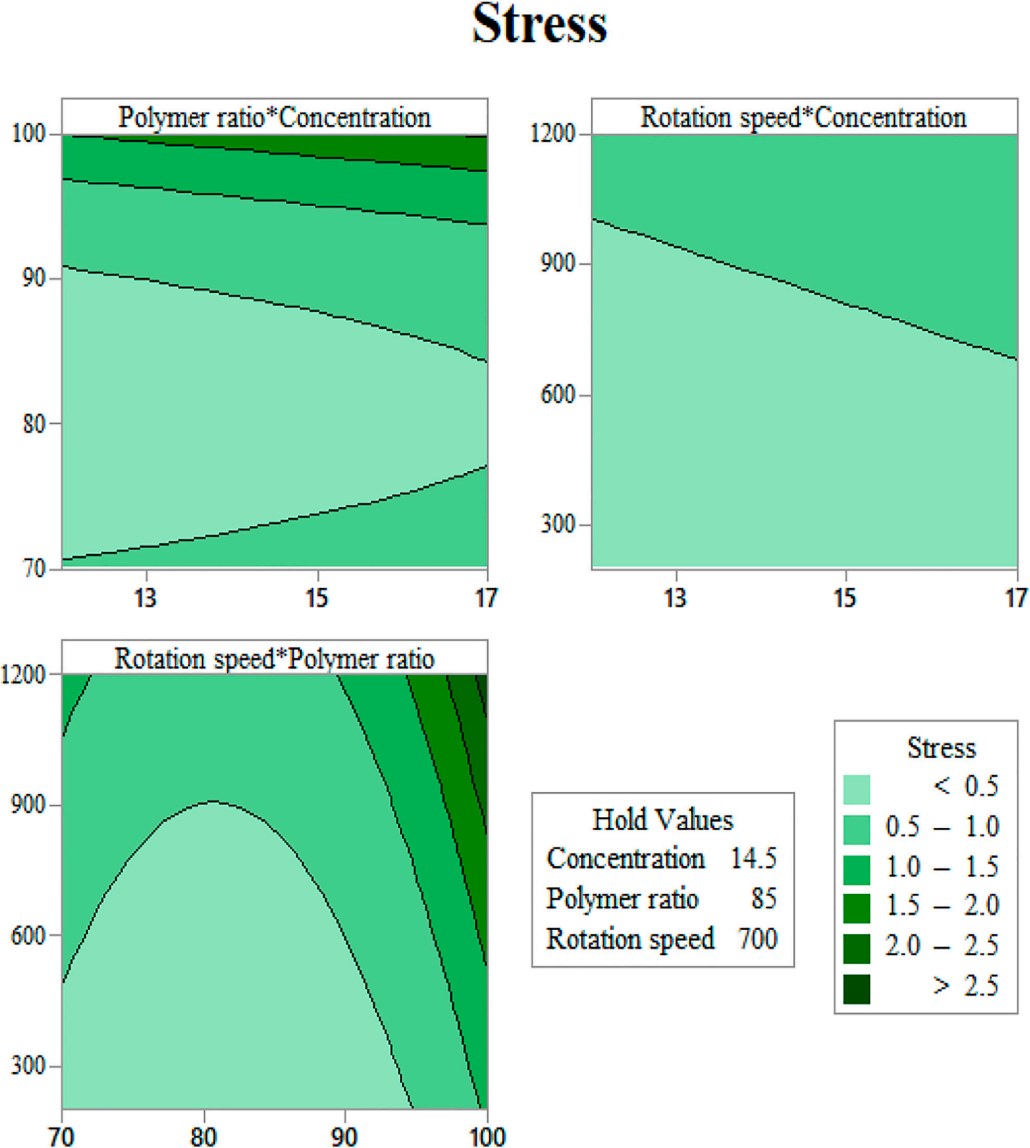
Yield stress contours as functions of input variables: **(A)** gelation content vs. concentration, **(B)** mandrel rotation speed vs. concentration, and **(C)** mandrel rotation speed vs. gelatin content.

In order to use visual aids for a better understanding of the results, the contour of the responses versus the variables, which are polymer ratio, concentration, and rotation speed, is plotted in Figures 4–6, all of which are the results of the fitted model based on the considered parameters. Figures 4–6 illustrate different values of the variables as a function of the response. In other words, the responses are displayed in the desired 2D cut planes of the variable. By use of these diagrams, the responses can be evaluated in a continuous manner. As such, the results of ANOVA and model summary of the three parameters of strain, stress, and modulus are tabulated in Tables 4–6. In these tables, degrees of freedom (DFs) represent the amount of info in the data, adjusted mean squares (Adj MS) calculate a variation of a term by considering all other terms in the model without paying attention to their order, and adjusted sums of squares (Adj SS) are the calculations of variation of different sources listed in the model (Almasvandi et al., 2016). R-squared (R-sq) and adjusted R-squared show the quality of the fitted model, and the predicted R-squared illustrates the efficiency of the model in terms of prediction with new inputs (Analysis of variance tabl, 2017). By considering polymer ratio as P, concentration as C, and rotation speed as R, the uncoded models for strain, stress, and modulus are shown in Eqs 4–6.

Strain^0.5 = 104.5 - 2.69 C - 1.609 P - 0.04567 R - 0.0226 C∗C + 0.00474 P∗P + 0.000005 R∗R +0.03773 C∗P + 0.000863 C∗R + 0.000303 P∗R, (4)

Stress^0.3 = 7.96 + 0.0213 C - 0.1923 P + 0.000329 R + 0.001191 P∗P, (5)

$$\begin{array}{l} \text{Ln}\left(\text{Modulus}\right)\;=\;-119\text{.}6\;+\;4\text{.}01\;\text{C}\;+\;1\text{.}836\;\text{P}\;+\;\text{0}\text{.}0492\;\text{R}\;-\;\text{0}\text{.}00646\;{\text{P}}^{*}\text{P}\;-\;\text{0}\text{.}000005\;{\text{R}}^{*}\text{R}\;-\\ \text{0}\text{.}0375\;{\text{C}}^{*}\text{P}\;-\;\text{0}\text{.}001341\;{\text{C}}^{*}\text{R}\;-\;\text{0}\text{.}000249\;{\text{P}}^{*}\text{R} \end{array}$$ (6)

**TABLE 4:** Analysis of variance for strain.

Source	DF	Adj SS	Adj MS	F-value	*p*-value
Model	9	38.5415	4.2824	13.21	0.003
Linear	3	16.5786	5.5262	17.04	0.002
Concentration	1	11.4025	11.4025	35.16	0.001
Polymer ratio	1	3.2926	3.2926	10.15	0.019
Rotation speed	1	0.1486	0.1486	0.46	0.524
Square	3	5.5612	1.8537	5.72	0.034
Concentration*Concentration	1	0.0550	0.0550	0.17	0.695
Polymer ratio*Polymer ratio	1	1.0452	1.0452	3.22	0.123
Rotation speed*Rotation speed	1	4.4673	4.4673	13.78	0.010
Two-way interaction	3	14.6980	4.8993	15.11	0.003
Concentration*Polymer ratio	1	5.4366	5.4366	16.77	0.006
Concentration*Rotation speed	1	1.8319	1.8319	5.65	0.055
Polymer ratio*Rotation speed	1	14.0533	14.0533	43.34	0.001
Error	6	1.9457	0.3243		
Total	15	40.4871			
Model Summary					
S	R-sq	R-sq (adj)		R-sq (pred)	
0.569,454	95.19%	87.99%		46.65%	

**TABLE 5:** Analysis of variance for stress.

Source	DF	Adj SS	Adj MS	F-value	*p*-value
Model	4	0.71843	0.17961	5.61	0.010
Linear	3	0.49144	0.16381	5.12	0.019
Concentration	1	0.02685	0.02685	0.84	0.379
Polymer ratio	1	0.20903	0.20903	6.53	0.027
Rotation speed	1	0.25556	0.25556	7.99	0.016
Square	1	0.22699	0.22699	7.10	0.022
Polymer ratio*Polymer ratio	1	0.22699	0.22699	7.10	0.022
Error	11	0.35187	0.03199		
Total	15	1.07030			
Model Summary					
S	R-sq	R-sq (adj)		R-sq (pred)	
0.178,851	67.12%	55.17%		34.36%	

**TABLE 6:** Analysis of variance for modulus.

Source	DF	Adj SS	Adj MS	F-value	*p*-value
Model	8	30.2627	3.7828	4.20	0.037
Linear	3	4.6855	1.5618	1.73	0.247
Concentration	1	0.7670	0.7670	0.85	0.387
Polymer ratio	1	0.6413	0.6413	0.71	0.427
Rotation speed	1	3.1063	3.1063	3.45	0.106
Square	2	6.7054	3.3527	3.72	0.079
Polymer ratio*Polymer ratio	1	1.9405	1.9405	2.16	0.186
Rotation speed*Rotation speed	1	4.7749	4.7749	5.30	0.055
Two-way Interaction	3	12.0413	4.0138	4.46	0.047
Concentration*Polymer ratio	1	5.3777	5.3777	5.97	0.045
Concentration*Rotation speed	1	4.4259	4.4259	4.92	0.062
Polymer ratio*Rotation speed	1	9.4981	9.4981	10.55	0.014
Error	7	6.3027	0.9004		
Total	15	36.5654			
Model Summary					
S	R-sq	R-sq (adj)		R-sq (pred)	
0.948,890	82.76%	63.06%		24.71%	

## Discussion

The scaffolds are designed and fabricated in order to mimic different characteristics of the original tissue. Mechanical and morphological characteristics of the scaffolds, as the main objectives of this study, are two essential items.

A challenge that bioengineers face is to employ the input variables of the electrospinning process, including the solution properties and the machine setup so as to achieve the desired mechanical strength. A large range of mechanical properties could be attained using the electrospinning method. Therefore, choosing the proper entry to this method can lessen the number of needed samples to be fabricated with appropriate mechanical and morphological properties. In this study, we put efforts to anticipate the morphological and mechanical behavior of Gel/CA scaffolds. We investigated the solution concentration, polymer weight ratio, and mandrel rotation speed as the input variables and their effect on elastic modulus, yield stress, and the rupture strain to describe them as functions of the input variables. Similarly, the alignments of fibers in the fabricated scaffolds were described by introducing α and *ß*, which represent the quantity of the aligned fibers and the distribution of the fibers, respectively. Experiments were designed by a Taguchi L′16 orthogonal array for three input variables with four levels. This design was chosen to reduce the number of tests to 16 instead of 64, and the combination of all 16 is listed in Table 1.

Considering fiber diameter values in Table 2 and mechanical properties in Table 3, we can find a good relationship between fiber diameter and Young’s modulus of the scaffold. This relation would be related to stronger bonding of the polymers that resulted in a large diameter of the fibers as well as resisting tensile loading to be ruptured. This relation was also observed in yield stress, which would approve the previous theory. On the other hand, strain at rupture decreases, with elevation of fiber diameter in the scaffold. This effect might lessen the total effective area for resisting tensile forces due to concentration of polymers in a single fiber instead of a highly distributed network.

As shown in Figures 2, 4, increasing the mandrel rotation speed has the most significant impact on the fiber alignment. It improves both the α and *β* parameters simultaneously. This is due to tension applied by the mandrel, which has a direct relation with its rotation speed. Following this tension, the fibers become more aligned and focused on the dominant direction. Accepting rotation speed as the first priority in the fiber alignments, the solution concentration and polymer weight ratio could have a minor effect on the fiber alignment. Figure 2B shows that the minimum of α occurs in scaffolds with an 80% weight ratio of Gel, while the maximum of α will take place at 70% of Gel content. This enhancement in α might be a result of increasing the viscosity of the solution, which prevents fiber breakage while spinning that overcomes the partial entanglement of two polymeric chains considering that the acetate group is hard to get entangled. *β* is influenced by the other input, that is, solution concentration. Increasing the concentration has two opposing effects on the solution. The increase in concentration leads to the higher viscosity of the solution, while it enlarges the solution surface tension. As shown in Figure 2C, These opposing factors meet at the optimum point of 13% to have a focused distribution of the fibers around the dominant direction. The results help us to classify the effective parameters to major and minor, where mandrel rotation speed has the major role in aligning the fibers according to both α and *ß* calculations. However, the role of the polymer weight ratio on the number of aligned fibers and solution concentration on reducing fiber deviation from the dominant direction as minor effective parameters could not be ignored. A quick look at the results of mechanical tests describes a complex system with a vast interaction between parameters. We applied the response surface method on the results to have a better understanding of how parameters influence mechanical characteristics.

Figure 3 and Table 3 illustrate mechanical properties of each experiment. Adding CA to gelatin solution at low concentrations reduced the strength of the scaffolds but enhanced the ductility of the fibrous sheet. At higher concentrations, where the chance of polymer bonding increased, more CA caused better mechanical strength as well as higher strain at rupture, which means higher ductility in the scaffolds.

Figure 4 indicates the elastic modulus of the fabricated scaffolds as functions of the input variables. Figure 4A illustrates the effect of polymer weight ratio and solution concentration on elastic modulus. As could be noticed, in low concentrations, elastic modulus increases with Gel amount. This might happen due to the increased surface tension, which has more impacts when the viscosity of the solution is low. High surface tension while the viscosity is low increases the probability of electrospraying. This tendency to spray instead of spin could result in losing uniformity in spun fibers. This type of defects in the scaffold structure could reduce the resistance of the scaffold against tensile force and, consequently, reduce the elastic modulus. However, in high concentrations, the scaffold behavior is the opposite. This alteration in the modulus trend is a response to increasing the viscosity and eliminates the surface tension effects. Increasing both the concentration and CA increases the viscosity; thus, the electrospun fibers are more stable and firmer.

**FIGURE 7:**
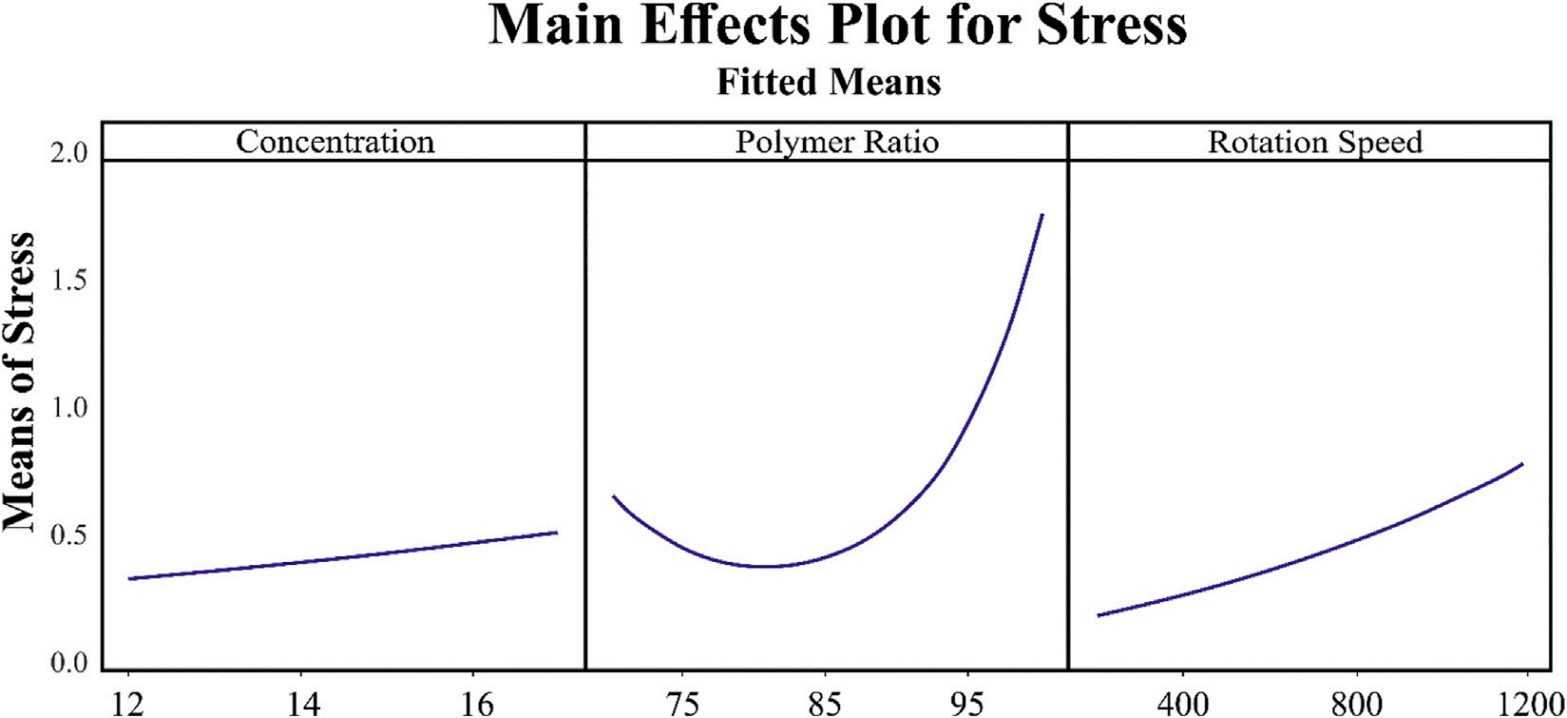
Relation of the main parameters, concentration, polymer ratio, and rotation speed, with stress.

Since in the generated formula for stress, the interaction of parameters is not involved, the main effect plot for each item is of equal, if not greater, importance. Based on Figure 7, the relation between solution concentration and mandrel rotation speed is linear, where the more the value, the more the stress, while for polymer ratio, it is nonlinear. For a value less than 82, the increase in polymer ratio leads to a decrease in stress, whereas for a value more than 82, an increase in polymer ratio results in an increase in polymer ratio.

Figure 4B illustrates the significant effect of mandrel rotation speed on elastic modulus. This parameter initiates its role since the solution leaves the tailor cone at the tip of the nozzle; therefore, it has no mark on fiber composition. The consequence of varying the rotation speed is on the scaffold structure. As is known, a higher speed of the mandrel causes the fibers to get aligned, and the aligned fibers need more stress to deform. Altogether it would be understood from the elastic modulus contours that an optimization between surface tension and viscosity will contribute to gain the higher modulus.

Determination of the scaffold strain at the rupture with respect to input variables is presented in Figure 5. It is easy to find out that Gel content has a direct relation with rupture strain (Figure 5A). This may be the reason of the gelation feature of gelatin, which let the scaffold to endure higher degrees of strain before breakage. By increasing the solution concentration, the fibers grow in diameter, and the strain becomes higher (Figure 5B). It should be noted that these scaffolds have a layer structure. Strain at rupture was measured when all the layers ruptured, and this layer-by-layer rupture enlarges the displacement before the total breakage. Mandrel rotation speed, which controls the fiber alignment, influences the amount of strain as well. The aligned fibers mean that there will be a greater number of fibers that resist tensile force rather than shear force (Figure 5). Considering the fibers as cables, they can resist great shear forces and will be easily cut.

As seen in Figure 6A, at a constant rotation speed of the mandrel, the yield stress drops with adding CA to the solution up to 20% and increases again after this value. This may be justified with the interaction between surface tension and viscosity. Adding a small amount of CA in substitution of Gel induces a jump in surface tension, while the increase in viscosity will be softer. As more CA is added, the slope of the rise in surface tension becomes milder, and viscosity can compensate for its slow improvement.

**FIGURE 8:**
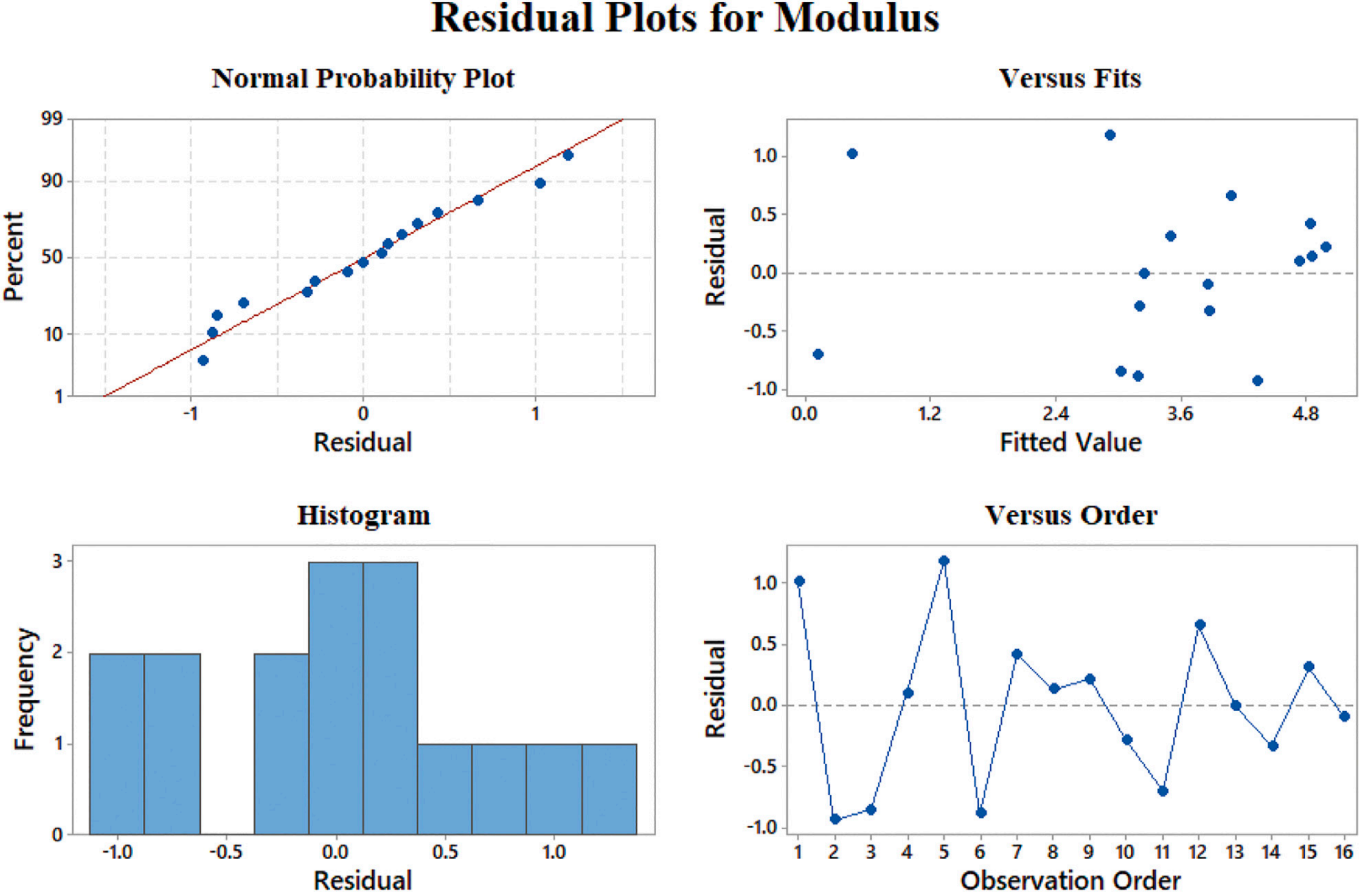
Representing errors of the elastic modulus model: **(A)** normal probability, **(B)** versus fit, **(C)** histogram, and **(D)** versus order.

In Figure 8A, the fitted curve for normal probability in elastic modulus results becomes a straight line, which approves the constant variance hypothesis on which we built our model. Figure 8B shows the results versus fitted values; the model has the best performance in 0–50 MPa and 100–130 MPa for elastic modulus since the least residuals exist in these ranges. The histogram for modulus results indicates that the most dominant quantity of fitted values has less than 10 MPa difference with experimental results, although we are able to find out that the largest amount of error occurs in the samples which have a much smaller elastic modulus related to the average rather than the samples with the ones with a modulus much larger than the average. The random pattern of the residuals in Figure 8D suggests the independence of data.

**FIGURE 9:**
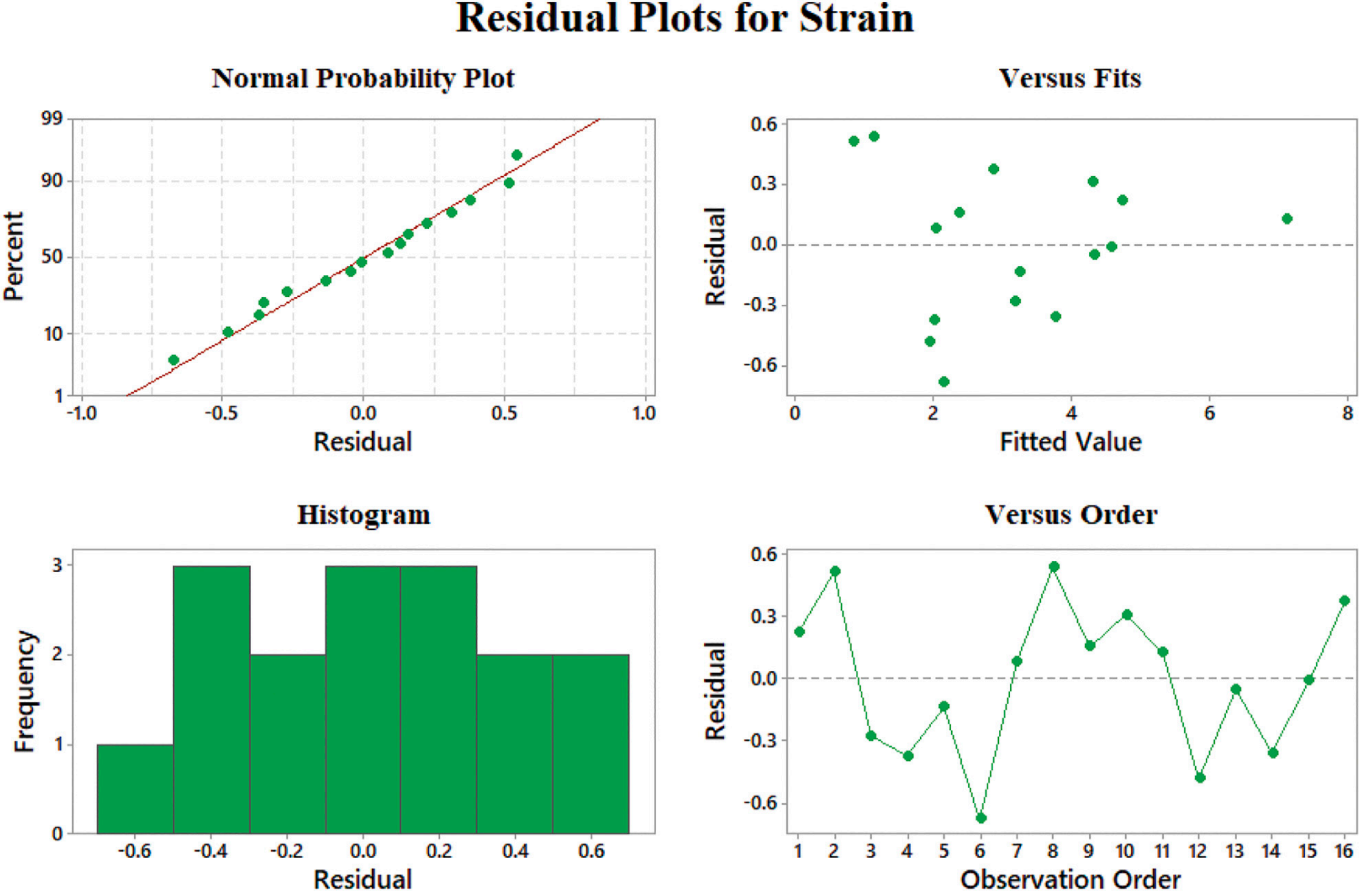
Representing errors of the strain at rupture model: **(A)** normal probability, **(B)** versus fit, **(C)** histogram, and **(D)** versus order.

Normal probability of the strain model (Figure 9A) shows no skewness in the results, while the best performance in the modeling of the strain at rupture data is 12–24% elongation according to Figure 9B. The residuals versus fitted values lower than 12% show fluctuation and may not be convincing in this range. Additionally, the histogram of this model shows that most of the data have less than 3% of residuals which admit the good behavior of our model. Besides, large negative residuals are more abundant in comparison with large positives. This means that our model can predict data with larger values properly.

**FIGURE 10:**
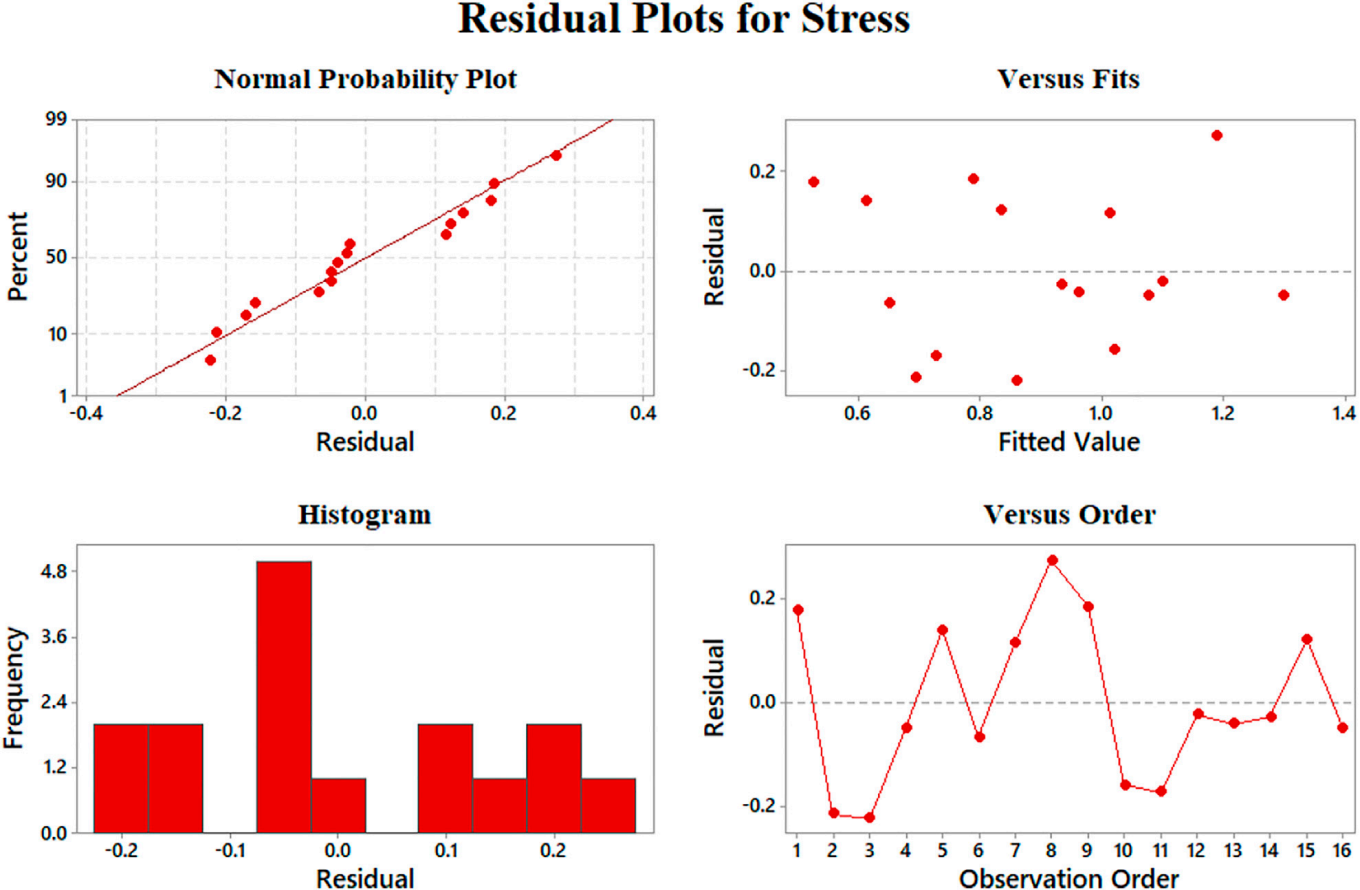
Representing errors of the yield stress model: **(A)** normal probability, **(B)** versus fit, **(C)** histogram, and **(D)** versus order.

Data aggregation around residual 0 means the proper modeling of stress results according to Figure 10A. The best prediction of the model belongs to 0.5–1 MPa, where the least value of residuals was achieved. On the other hand, the errors will grow beyond the yield stress of 2 MPa. We could claim that none of our results is an outlier regarding Figures 8D, 9D, 10D since there is no sample with a great error value. There is no skewness in our data sets, and almost in all the values of elastic modulus, strain at rupture, and yield stress, the model has a reasonable difference with experimental values.

## Conclusion

In this study, we attempted to define the interaction between process variables in the electrospinning method and their effect on the structural and mechanical properties of the scaffolds fabricated in this study. The major step that we took in this study was to define a model which simultaneously considers Young’s modulus, yield stress, and strain at rupture as inputs and shows the engineers the suitable fabrication values, polymer weight ratio, solution concentration, and mandrel rotation speed, in the electrospinning method. All three mentioned mechanical properties play a significant role in the behavior of the engineered scaffold. Thus, knowing the values for the fabrication parameters will contribute to decreasing the time and material consumption for engineers. For this to happen, we designed and fabricated 16 different scaffolds with an individual composition of input variables and observed them under SEM to determine their morphological and mechanical characteristics. The alignments of the fibers were quantified by defining two parameters, which are α and *β*, and the effect of each input on fiber alignment was discussed. Mechanical characteristics of the scaffolds, elastic modulus, yield stress, and strain at rupture, were measured by uniaxial mechanical testing. The experimental outputs were modeled with the RSM method. Thereupon, continuous prediction of how parameters affect the mechanical properties was figured and discussed. Finally, the probability of the model and experimental results were examined.

## Data Availability

The original contributions presented in the study are included in the article/Supplementary Material, and further inquiries can be directed to the corresponding authors.
